# Olprinone Attenuates the Acute Inflammatory Response and Apoptosis after Spinal Cord Trauma in Mice

**DOI:** 10.1371/journal.pone.0012170

**Published:** 2010-09-07

**Authors:** Emanuela Esposito, Emanuela Mazzon, Irene Paterniti, Daniela Impellizzeri, Placido Bramanti, Salvatore Cuzzocrea

**Affiliations:** 1 Department of Clinical and Experimental Medicine and Pharmacology, School of Medicine, University of Messina, Messina, Italy; 2 IRCCS Centro Neurolesi “Bonino-Pulejo”, Messina, Italy; Julius-Maximilians-Universität Würzburg, Germany

## Abstract

**Background:**

Olprinone hydrochloride is a newly developed compound that selectively inhibits PDE type III and is characterized by several properties, including positive inotropic effects, peripheral vasodilatory effects, and a bronchodilator effect. In clinical settings, olprinone is commonly used to treat congestive cardiac failure, due to its inotropic and vasodilating effects. The mechanism of these cardiac effects is attributed to increased cellular concentrations of cAMP. The aim of the present study was to evaluate the pharmacological action of olprinone on the secondary damage in experimental spinal cord injury (SCI) in mice.

**Methodology/Principal Findings:**

Traumatic SCI is characterized by an immediate, irreversible loss of tissue at the lesion site, as well as a secondary expansion of tissue damage over time. Although secondary injury should be preventable, no effective treatment options currently exist for patients with SCI. Spinal cord trauma was induced in mice by the application of vascular clips (force of 24 g) to the dura via a four-level T5–T8 laminectomy. SCI in mice resulted in severe trauma characterized by edema, neutrophil infiltration, and production of inflammatory mediators, tissue damage, apoptosis, and locomotor disturbance. Olprinone treatment (0.2 mg/kg, i.p.) 1 and 6 h after the SCI significantly reduced: (1) the degree of spinal cord inflammation and tissue injury (histological score), (2) neutrophil infiltration (myeloperoxidase activity), (3) nitrotyrosine formation, (4) pro-inflammatory cytokines, (5) NF-κB expression, (6) p-ERK1/2 and p38 expression and (7) apoptosis (TUNEL staining, FAS ligand, Bax and Bcl-2 expression). Moreover, olprinone significantly ameliorated the recovery of hind-limb function (evaluated by motor recovery score).

**Conclusions/Significance:**

Taken together, our results clearly demonstrate that olprinone treatment reduces the development of inflammation and tissue injury associated with spinal cord trauma.

## Introduction

Olprinone is a selective phosphodiesterase type III (PDE III) inhibitor widely used to treat circulatory conditions. Olprinone is clinically used for treatment of acute heart failure [1], [2] or cerebral ischemia [3]. Olprinone was originally developed as a cardiotonic agent, having positive inotropic and vasodilator actions. It improves myocardial mechanical efficiency [4] via elevation of intracellular cyclic AMP (cAMP) levels in both cardiomyocytes and vascular smooth muscle cells. It also increases myocardial contractility and reduces vascular resistance, leading to an improvement of hemodynamic status [5]. Moreover, olprinone augments cerebral blood flow by its direct vasodilator effect on the cerebral arteries. Olprinone inhibits vascular contractility by decreasing cytosolic Ca^2+^ levels and the Ca^2+^ sensitivity of the contractile elements: these effects may be mediated by an increase in cAMP content [6]. Finally, olprinone inhibits both von Willebrand factor mediated and fibrinogen mediated platelet aggregation [7]. In addition, olprinone has anti-inflammatory actions at therapeutic concentrations clinically used for heart failure [8].

Spinal cord injury (SCI) is a highly debilitating pathology [9]. Although innovative medical care has improved patient outcome, advances in pharmacotherapy for the purpose of limiting neuronal injury and promoting regeneration have been limited. Traumatic SCI causes severe and permanent functional deficits due to the primary mechanical insult followed by secondary tissue degeneration which may take which may take place over a period of weeks or even months [10]. A key mediator of this process is an acute and robust inflammatory response which involves the synthesis and release of chemo- and cytokines and a coordinated recruitment of circulating leucocytes as well as microglia from the CNS parenchyma [11], [12], [13], [14].

A recent report indicates that increased intracellular cAMP levels lead to reduced SCI [15]. Increased intracellular cAMP levels also reduce ischemia/reperfusion (IR)-induced renal injury [16], [17], probably due to reduced neutrophil accumulation [18]. No reports have addressed the role for olprinone in pathophysiology of SCI. Interestingly, olprinone reduces the IR-induced acute renal injury in rats through enhancement of cAMP, inhibition of leukocyte activation [19], and decreasing elevated concentrations of cytokine-induced neutrophil chemoattractant-1 in septic rats [20].

No reports have addressed a role for olprinone in pathophysiology of SCI.

In this study, the use of olprinone hydrochloride, a specific PDE III inhibitor, allowed us to demonstrate that PDE III activation plays a key role in the modulation of secondary injury in the spinal cord. In particular, we evaluated for the first time the effects of olprinone, determining 1) the extent of anatomical injury, 2) hind limb motor function 10 days after injury, 3) concentrations of key pro-inflammatory cytokines that are important mediators of secondary injury within the spinal cord, (4) neutrophil infiltration, (5) NF-κB expression, (6) MAP kinases expression, (7) nitrotyrosine formation, (8) apoptosis as TUNEL staining, (9) the Bcl-2 family of proteins, and (10) S100β expression.

The present manuscript is a study aimed at demonstrating that olprinone administration after experimental SCI in mice inhibits pathways leading to inflammation and apoptotic cell death and has a positive effect on the neurological outcome. Considering that initiation of inflammatory responses in CNS is related to activation of MAPKs, especially ERK1/2 and p38 MAPK, and that their activation would be a determinant for neuronal death or survival on certain occasions, the pharmacological impact of olpirone on spinal cord trauma is of interest to medical scientists.

## Materials and Methods

### Animals

Male adult CD1 mice (25–30 g, Harlan Nossan, Milan, Italy) were used for all studies. Mice were housed in individual cages (5 for each group) and maintained under 12∶12 light-dark cycle at 21±1°C and 50±5% humidity. The animals were acclimated to their environment for 1 wk and had *ad libitum* access to tap water and standard rodent standard diet. All animal experiments complied with regulations in Italy (D.M. 116192), Europe (O.J. of E.C. L 358/1 12/18/1986) and USA (Animal Welfare Assurance No A5594-01, Department of Health and Human Services, USA). All behavioral testing was conducted in compliance with the NHI laboratory animal care guidelines and with protocols approved by the Institutional Animal Care and Use Committee (Council directive # 87-848, October 19, 1987, Ministère de l'Agriculture et de la Forêt, Service Vétérinaire de la Santé et de la Protection Animale, permission # 92-256 to SC). The study was approved by the University of Messina Review Board for the care of animals.

### Spinal cord injury

Mice were anaesthetized using chloral hydrate (400 mg/kg body weight). We used the clip compression model described by Rivlin and Tator [21] and produced SCI by extradural compression of spinal cord section exposed via a four-level T5–T8 laminectomy, in which the prominent spinous process of T5 was used as a surgical guide. With the aneurysm clip applicator oriented in the bilateral direction, an aneurysm clip with a closing force of 24 g was applied extradurally at T5–T8 level. The clip was then rapidly released with the clip applicator, which caused SC compression. In the injured groups, the cord was compressed for 1 min. Following surgery, 1.0 cc of saline was administered subcutaneously in order to replace the blood volume lost during the surgery. During recovery from anesthesia, the mice were placed on a warm heating pad and covered with a warm towel. Food and water were provided to the mice ad libitum. During this time period, the animals' bladders were manually voided twice a day until the mice were able to regain normal bladder function. Sham injured animals were only subjected to laminectomy. Spinal cord tissues were taken at 24 h following trauma. Tissue segments contained the lesion (1 cm on each side of the lesion), in according to counts of retrogradely labeled neurons at the origin of distinct descending motor pathways and to morphometric assessments of normal residual tissue at the injury epicenter, as previously described by Joshi and Fehlings [22].

### Experimental Design

Mice were randomized into 4 groups (N = 40 animals/group). Sham animals, subjected to the surgical procedure except that the aneurysm clip was not applied, were treated intraperitoneally (i.p.) with vehicle (saline) or olprinone (0.2 mg/kg) 1 h and 6 h after surgical procedure. The remaining mice were subjected to SCI (as described above) and treated with an ip. bolus of vehicle (saline) or olprinone (0.2 mg/kg) 1 h and 6 h after SCI. The doses of olprinone (0.2 mg/kg) used here were based on previous *in vivo* studies [23], [24] and in agreement with preliminary dose-response study (0.05–0.2 mg/kg). In experiments on motor score, the animals were treated with olprinone (0.2 mg/kg), 1 h and 6 h after SCI and daily until day 9. Ten mice from each group were sacrificed at different time points in order to collect samples for the evaluation of different parameters. Olprinone hydrochloride (SIGMA-ALDRICH) was dissolved in normal saline and administered ip.

### Myeloperoxidase activity

Myeloperoxidase (MPO) activity, an indicator of polymorphonuclear leukocyte (PMN) accumulation, was determined in the spinal cord tissues as previously described [25] at 24 hours after SCI. At the specified time following SCI, spinal cord tissues were obtained and weighed, each piece homogenized in a solution containing 0.5% (w/v) hexadecyltrimethyl-ammonium bromide dissolved in 10 mM potassium phosphate buffer (pH 7) and centrifuged for 30 min at 20,000× g at 4°C. An aliquot of the supernatant was then allowed to react with a solution of 1.6 mM tetramethylbenzidine and 0.1 mM H_2_O_2_. The rate of change in absorbance was measured spectrophotometrically at 650 nm. MPO activity was defined as the quantity of enzyme degrading 1 µmol of peroxide per min at 37°C and was expressed as units of MPO/g wet tissue.

### Immunohistochemical localization of P-selectin, Intercellular Cell Adhesion Molecule (ICAM)-1, IL-1 β, TNF-α, nitrotyrosine, S-100, PAR, Fas Ligand (FasL), Bax and Bcl-2

Twenty-four hours after SCI, the tissues were fixed in 10% (w/v) PBS-buffered formaldehyde and 8 µm sections were cut from paraffin embedded tissues and mounted on positively charged slides for immunohistochemical staining. After deparaffinization, endogenous peroxidase was quenched with 0.3% (v/v) hydrogen peroxide in 60% (v/v) methanol for 30 min. The sections were permeabilized with 0.1% (w/v) Triton X-100 in PBS for 20 min. Non-specific adsorption was minimized by incubating the section in 2% (v/v) normal goat serum in PBS for 20 min. Endogenous biotin or avidin binding sites were blocked by sequential incubation for 15 min with biotin and avidin, respectively. Sections were incubated overnight with anti-GFAP (Cell Signaling, 1∶500), anti-P-selectin antibody (Pharmingen, 1∶500 in PBS, v/v), anti-ICAM-1 antibody (Pharmingen,1∶500, in PBS, v/v), anti-nitrotyrosine rabbit polyclonal antibody (Upstate, 1∶500 in PBS, v/v), anti-PAR antibody (BioMol, 1∶200 in PBS, v/v), anti-FasL antibody (Santa Cruz Biotechnology, 1∶500 in PBS, v/v), anti-TNF-α ligand antibody (Santa Cruz Biotechnology, 1∶500 in PBS, v/v), anti-IL-1β ligand antibody (Santa Cruz Biotechnology, 1∶500 in PBS, v/v), anti-Bax antibody (Santa Cruz Biotechnology, 1∶500 in PBS, v/v) or with anti-Bcl-2 polyclonal antibody (Santa Cruz Biotechnology, 1∶500 in PBS, v/v) or with anti-S-100 polyclonal antibody (Santa Cruz Biotechnology, 1∶500 in PBS, v/v). Sections were washed several times with PBS, incubated with a biotinylated goat anti-rabbit secondary antibody and in avidin-biotin peroxidase complex (Vectastatin, ABC elite kit, Vector Laboratories).

In order to confirm that the immunoreaction for the nitrotyrosine was specific some sections were also incubated with the primary antibody (anti-nitrotyrosine) in the presence of excess nitrotyrosine (10 mM) to verify the binding specificity. To verify the binding specificity for IL-1β, TNF-α, PAR, FasL, Bax and Bcl-2, some sections were also incubated with only the primary antibody (no secondary) or with only the secondary antibody (no primary). In these situations no positive staining was found in the sections indicating that the immunoreactions were positive in all the experiments carried out.

Immunocytochemistry photographs (n = 5) were assessed by densitometry and corrected to the background signal. Background settings were adjusted from examination of negative control specimens. The assay was carried out by using Optilab Graftek software on a Macintosh personal computer (CPU G3-266). All the immunocytochemistry analysis was carried out without knowledge of the treatments.

### Terminal Deoxynucleotidyltransferase-Mediated UTP End Labeling (TUNEL) Assay

TUNEL assay was conducted by using a TUNEL detection kit according to the manufacturer's instruction (Apotag, HRP kit DBA, Milan, Italy). Briefly, sections were incubated with 15 µg/ml proteinase K for 15 min at room temperature and then washed with PBS. Endogenous peroxidase was inactivated by 3% H_2_O_2_ for 5 min at room temperature and then washed with PBS. Sections were immersed in terminal deoxynucleotidyltransferase (TdT) buffer containing deoxynucleotidyl transferase and biotinylated dUTP in TdT buffer, incubated in a humid atmosphere at 37°C for 90 min, and then washed with PBS. The sections were incubated at room temperature for 30 min with anti-horseradish peroxidase-conjugated antibody, and the signals were visualized with diaminobenzidine. The number of TUNEL positive cells/high-power field was counted in 5 to 10 fields for each coded slide.

### Western blot analysis for IκB-α, NF-κB p65, Bax, Bcl-2, FasL, p-ERK1/2, and phospho-p38 (Thr180/Tyr182)

Cytosolic and nuclear extracts were prepared as previously described [26] with slight modifications. Briefly, spinal cord tissues from each mouse were suspended in extraction Buffer A containing 0.2 mM PMSF, 0,15 µM pepstatin A, 20 µM leupeptin, 1 mM sodium orthovanadate, homogenized at the highest setting for 2 min, and centrifuged at 1,000× g for 10 min at 4°C. Supernatants represented the cytosolic fraction. The pellets, containing enriched nuclei, were re-suspended in Buffer B containing 1% Triton X-100, 150 mM NaCl, 10 mM TRIS-HCl pH 7.4, 1 mM EGTA, 1 mM EDTA, 0,2 mM PMSF, 20 µm leupeptin, 0,2 mM sodium orthovanadate. After centrifugation 30 min at 15,000× g at 4°C, the supernatants containing the nuclear protein were stored at −80 for further analysis. The levels of IκB-α, p-ERK1/2, phospho-p38 MAP Kinase, Fas-L, Bax, and Bcl-2 were quantified in cytosolic fraction from spinal cord tissue collected after 24 hours after SCI, while NF-κB p65 levels were quantified in nuclear fraction.

Forty micrograms of protein was dissolved in Laemmli's sample buffer, boiled for 5 min, and subjected to SDSPAGE (8% or 15% polyacrylamide). The blot was performed by transferring proteins from a slab gel to nitrocellulose membrane at 240mA for 40 min at room temperature using TRANS-BLOT® SD Semy-dry Transfer Cell (BIORAD). The filters were blocked with 1x PBS, 5% (w/v) non fat dried milk (PM) for 40 min at room temperature and subsequently probed with specific Abs IκB-α (Santa Cruz Biotechnology, 1∶1000), or anti-Bax (1∶500; Santa Cruz Biotechnology), or anti-Bcl-2 (1∶500; Santa Cruz Biotechnology), or anti-pERK1/2 (1∶1000 Santa Cruz Biotechnology) or anti-NF-κB p65 (1∶1000; Santa Cruz Biotechnology) or anti-FasL (1∶200; Santa Cruz Biotechnology, C-178, IgG) or anti-phospho-p38 MAP Kinase (Thr180/Tyr182) (1∶1000; Cell Signaling) in 1x PBS, 5% w/v non fat dried milk, 0.1% Tween-20 (PMT) at 4°C, overnight. Membranes were incubated with peroxidase-conjugated bovine anti-mouse IgG secondary antibody or peroxidase-conjugated goat anti-rabbit IgG (1∶2000, Jackson ImmunoResearch, West Grove, PA) for 1 h at room temperature.

To ascertain that blots were loaded with equal amounts of proteic lysates, they were also incubated in the presence of the antibody against α-tubulin (for cytosolic extract) or lamin A/C (for nuclear extract) proteins (1∶10,000 Sigma-Aldrich Corp.).

Signals were detected with enhanced chemiluminescence detection system reagent according to manufacturer's instructions (SuperSignal West Pico Chemiluminescent Substrate, Pierce). The relative expression of the protein bands of IκB-α (∼37 kDa), NF-κB p65 (65 kDa), Bax (∼23 kDa), Bcl-2 (∼26 kDa), FasL (∼40 kDa), phospho-p38 MAP Kinase (43 kDa), was quantified by densitometry with Image Quant TL software (GE Healthcare) and standardized for densitometry analysis to housekeeping gene levels. The dual-phosphorylated form of ERK (p-ERK) antibody identified two bands of approximately 44 and 42 kDa (corresponding to p-ERK1 and p-ERK2, respectively).

Images of blot signals (8 bit/600 dpi resolution) were imported to analysis software (Image Quant TL, v2003). A preparation of commercially available molecular weight markers (Precision Plus Protein Kaleidoscope standards, BIO-RAD) consisting of proteins of molecular weight 10 to 250 kDa was used to define molecular weight positions and as reference concentrations for each molecular weight.

### Light microscopy

Spinal cord tissues were taken at 24 h following trauma. Tissue segments containing the lesion (1 cm on each side of the lesion) were paraffin embedded and cut into 5-µm-thick sections. Tissue sections (thickness 5 µm) were deparaffinized with xylene, stained with Haematoxylin/Eosin (H&E), and studied using light microscopy (Dialux 22 Leitz).

The segments of each spinal cord were evaluated by an experienced histopathologist. Damaged neurons were counted and the histopathologic changes of the gray matter were scored on a 6-point scale [27]: 0, no lesion observed, 1, gray matter contained 1 to 5 eosinophilic neurons; 2, gray matter contained 5 to 10 eosinophilic neurons; 3, gray matter contained more than 10 eosinophilic neurons; 4, small infarction (less than one third of the gray matter area); 5, moderate infarction; (one third to one half of the gray matter area); 6, large infarction (more than half of the gray matter area). The scores from all the sections from each spinal cord were averaged to give a final score for individual mice. All the histological studies were performed in a blinded fashion.

### ELISA measurement of TNF-α and IL-1β

For the measurement of cytokines levels, a 1 cm sample containing the lesion site (or comparable region of sham operated animals) was rapidly dissected and homogenized in 1 ml PBS containing protease inhibitors (Complete protease inhibitor tablets, Roche). TNF-α and IL-1β levels were assayed using DuoSet ELISA Development System (R&D Systems). All assays were carried out in duplicate using recommended buffers, diluents and substrates. Absorbency was determined using a microplate reader at 450 nm (Thermo Scientific, Multiskan FC Microplate Photometer). The intra-assay coefficient of variations for both assays was less than 10%. The concentration of the cytokines in the tissue was mentioned as pg/100 mg wet tissue.

### Quantification of BMS Score

The BMS locomotor rating scale analyzed seven locomotor categories. In our experiments, the motor function of mice subjected to compression trauma was assessed once a day for 10 days after injury. Recovery from motor disturbance was graded using the Basso Mouse Scale [28].

### Materials

Olprinone hydrochloride was obtained from SIGMA-ALDRICH (CAS Number: 119615-63-3). All compounds were from Sigma-Aldrich Company Ltd. (Milan, Italy). All other chemicals were of the highest commercial grade available. All stock solutions were prepared in non-pyrogenic saline (0.9% NaCl; Baxter, Italy, UK).

### Statistical evaluation

All values in the figures and text are expressed as mean ± standard error of the mean (SEM) of N observations. For the in vivo studies N represents the number of animals studied. In the experiments involving histology or immunohistochemistry, the figures shown are representative of at least three experiments performed on different experimental days. The results were analyzed by one-way ANOVA followed by a Bonferroni post-hoc test for multiple comparisons. A p-value of less than 0.05 was considered significant. BMS scale data were analyzed by the Mann-Whitney test and considered significant when p- value was <0.05.

## Results

### Olprinone reduces the severity of spinal cord trauma

**Figure 1:**
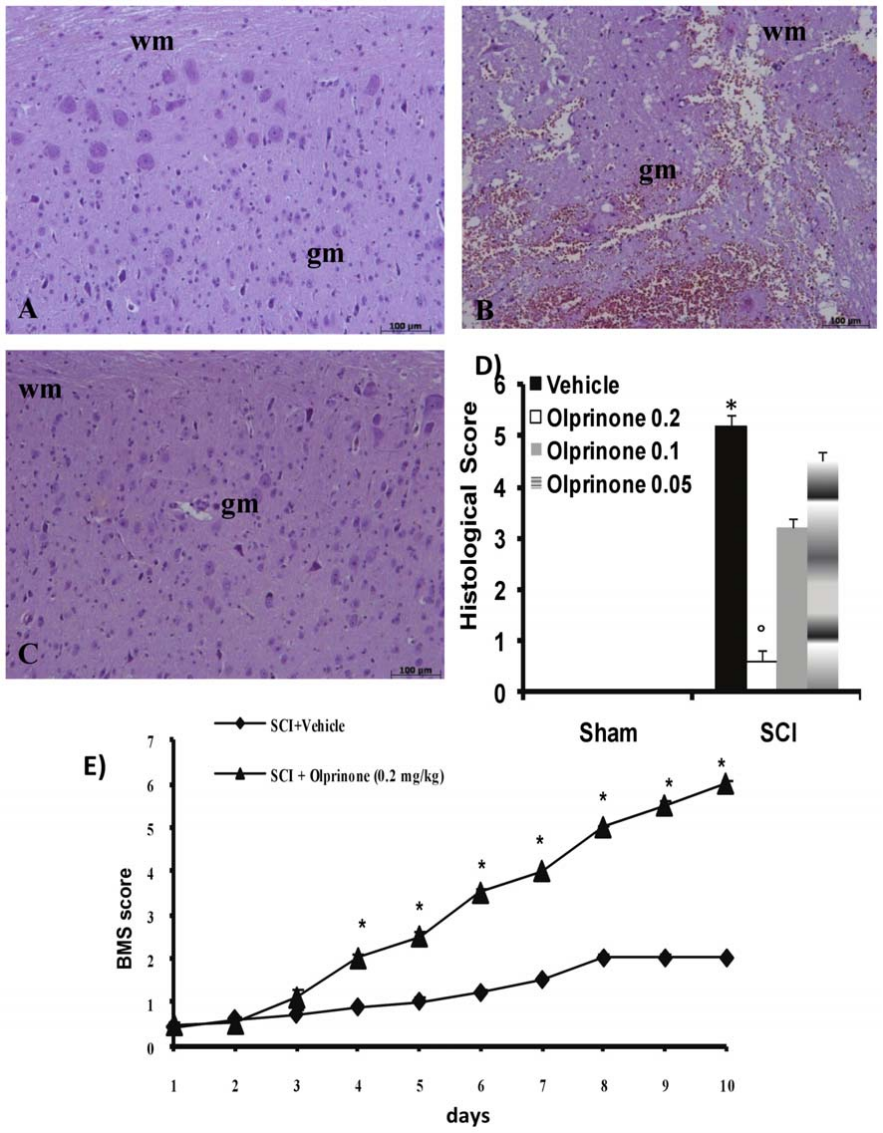
Effect of olprinone treatment on histological alterations of the spinal cord tissue 24 h after injury. A significant damage to the spinal cord at the perilesional area was assessed by the presence of edema as well as alteration of the white matter 24 h after injury (**B**). Notably, a significant protection from the SCI was observed in the tissue collected from olprinone treatment (0,2 mg/kg) treated mice (**C**). The histological score (**D**) was made by an independent observer and showed also a dose-response study with 0,05-0,1-0,2 mg/kg olprinone. wm: White matter; gm: gray matter. This figure is representative of at least 3 experiments performed on different experimental days. The degree of motor disturbance (**E**) was assessed every day until 10 days after SCI using the Basso Mouse Scale. Treatment with olprinone (0,2 mg/kg) reduces the motor disturbance after SCI. Values shown are mean ± s.e. mean of 10 mice for each group. °*p*<0.01 vs. *SCI*.

The severity of the trauma at the level of the perilesional area assessed as the presence of edema as well as alteration of the white matter (Figure 1B and see histological score D), was evaluated at 24 h after injury. A significant damage to the spinal cord was observed in the spinal cord tissue from SCI mice when compared with sham-operated mice (1B). Notably, a significant protection against the spinal cord injury was observed in injured mice treated with olprinone 0.2 mg/kg (Figure 1C and see histological score D). The dose of olprinone (0.2 mg/kg) used here were based on preliminary dose-response study (0.05–0.2 mg/kg), showing a significant protective effect of olprinone only at highest dose. In order to evaluate if histological damage to the spinal cord was associated with a loss of motor function, the modified BMS hind limb locomotor rating scale score was evaluated. While motor function was only slightly impaired in sham mice, mice subjected to SCI had significant deficits in hind limb movement (Figure 1E). Olprinone treatment ameliorated the functional deficits induced by SCI (Figure 1E).

### Olprinone reduces the activation of astrocytes after SCI

**Figure 2:**
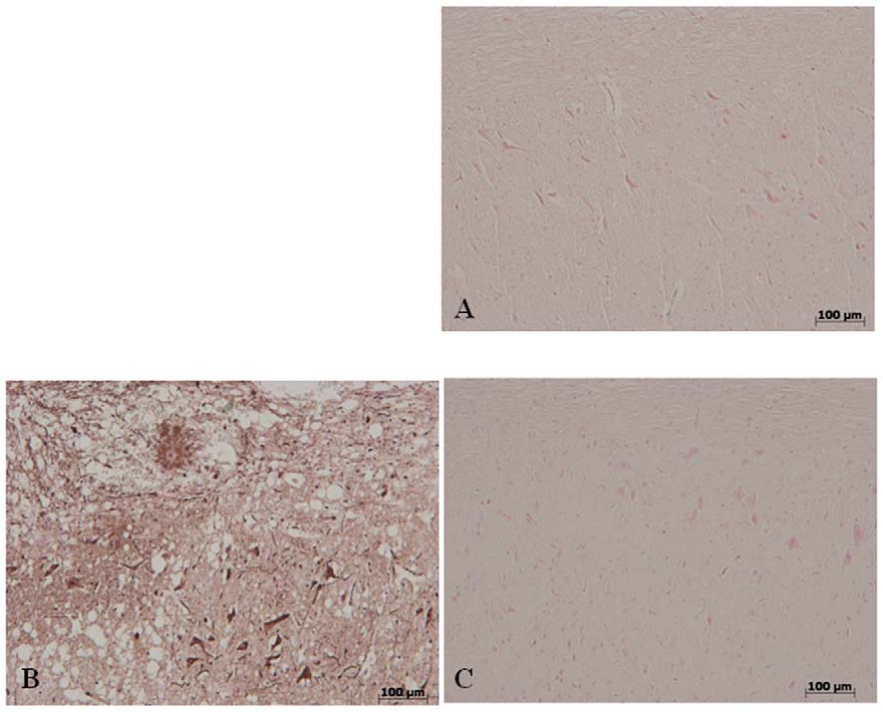
Effect of olprinone treatment on astrogliosis in the spinal cord tissue 24 h after injury. No positive staining for GFAP were observed in the spinal cord tissues collected from sham-operated mice (**a**). A significant presence of GFAP positive cells was found in the spinal cord, from SCI operated mice at the perilesional area 24 h after injury (**b**). Notably, a significant reduction of GFAP positive cells was observed in the tissue collected from olprinone-treated mice (**c**). This figure is representative of at least 3 experiments performed on different experimental days.

The cellular changes occurring in the perilesioned zone (namely, at the boundary between the core necrotic area and the penumbra zone) were evaluated by the expression of specific glial/precursor cell markers. In line with previous literature data [29], the characterization of spinal cord sections revealed increased astrogliosis (GFAP^+^ cells) in the perilesioned area from SCI animals in comparison to sham-operated animals (Figure 2B). On the contrary, significant less GFAP positive cells were found in the spinal cord tissues after SCI collected from mice which have been treated with olprinone (Figure 2C).

### Effects of olprinone on neutrophil infiltration and protein family of Cell Adhesion Molecules

**Figure 3:**
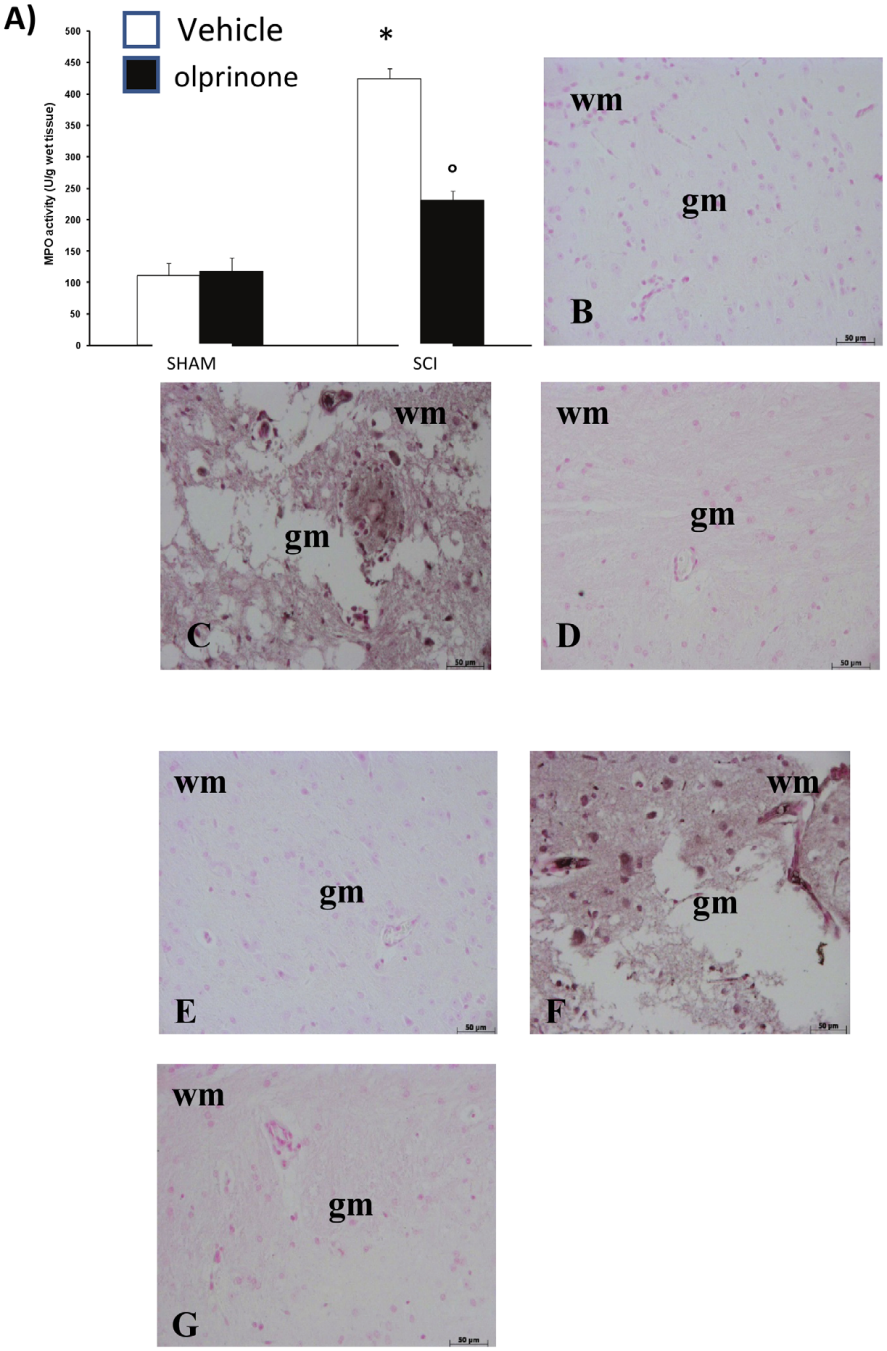
Effects of olprinone on MPO activity and superfamily of adhesion molecules. Following the injury, MPO activity in spinal cord from *SCI* mice was significantly increased at 24 h after the damage in comparison to sham groups (**A**). In addition, at 24 h after SCI, a positive immunohistochemical staining for ICAM-1 (**C**) and for P-selectin (**F**) was found in spinal cord sections compared to sham-operated mice (**B** and **E**, respectively). The positive immunostaining for ICAM-1 and for P-selectin was significantly decreased in sections from mice treated with olprinone (0,2 mg/kg) (D and G, respectively).

The abovementioned histological pattern of spinal cord injury appeared to be correlated with the influx of leukocytes into the spinal cord. Therefore, we investigated the effect of olprinone on the neutrophil infiltration by measuring tissue MPO activity. MPO activity was significantly elevated in the spinal cord at 24 h after injury in mice subjected to SCI when compared with sham-operated mice (Figure 3A). Treatment with olprinone attenuated neutrophil infiltration into the spinal cord at 24 h after injury (Figure 3A). Moreover, at 24 h after SCI, a positive immunohistochemical staining for ICAM-1 (Figure 3C; 6.50±0.1, p<vs. Sham, n = ) and for P-selectin (Figure 3F; 5.50±0.09, p<vs. Sham, n = ) was found in sections of spinal cord tissues compared to sham-operated mice. The positive immunostaining for ICAM-1 (Figure 3D; 0.17±0.09 vs. 6.50±0.1, p< vs. SCI, n = ) and for P-selectin (Figure 3G; 0.20±0.1 vs. 5.50±0.09, p< vs. SCI, n = ) was significantly decreased in sections from mice treated with olprinone (0,2 mg/kg).

### Olprinone modulate the expression of TNF-α and IL-1β after SCI

**Figure 4:**
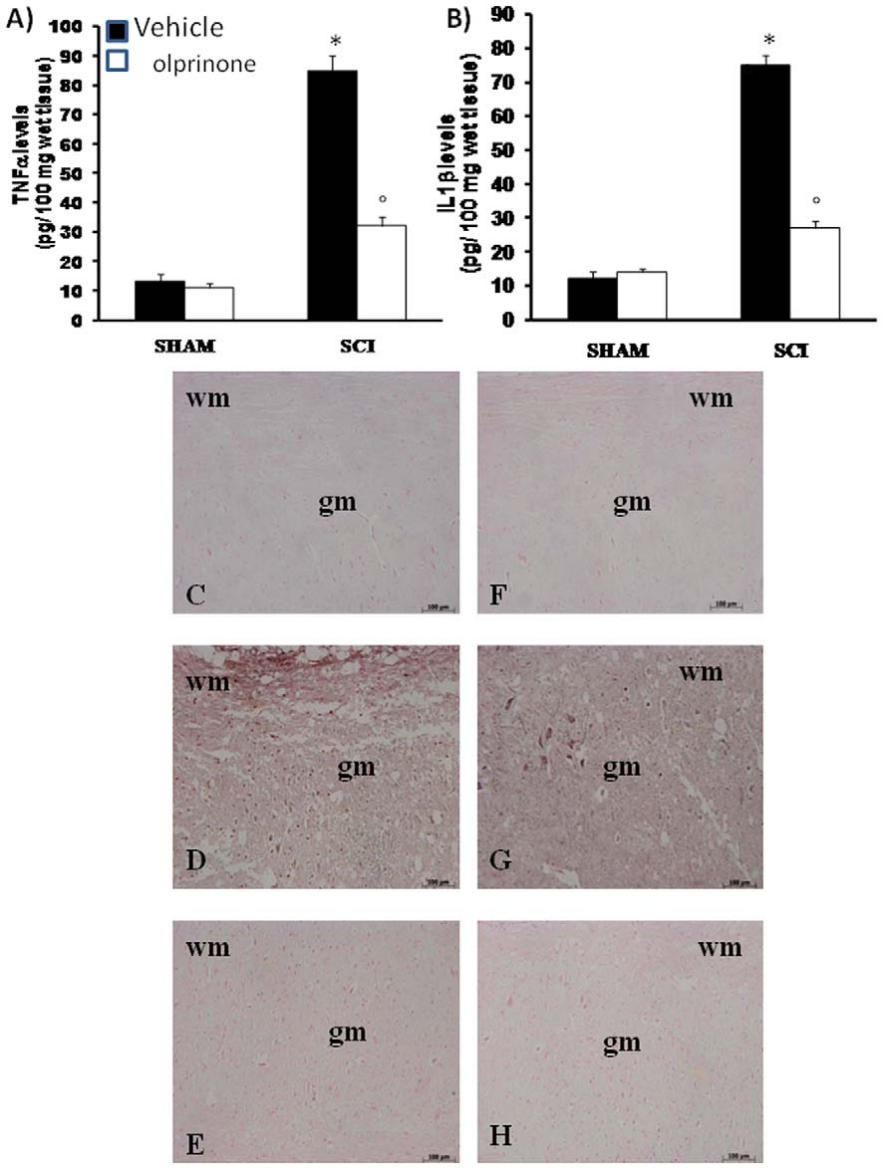
Effects of olprinone on pro-inflammatory cytokines. By ELISA measurement, TNF-α (**A**) and IL-1β (**B**) levels were significantly increased in the spinal cord from SCI mice. On the contrary, olprinone treatment (0,2 mg/kg 1 h and 6 h after SCI induction) prevented the SCI-induced levels of these cytokines (A and B). In addition, spinal cord sections were processed at 24 h after SCI to determine the immunohistological staining for TNF-α and IL-1β expression. A substantial increase in TNF-α (**D**) and IL-1β (**G**) expression was found in inflammatory cells, in wm and gm of the spinal cord tissues from SCI mice. Tissue expression of TNF-α (**E**) and IL-1β (**H**) were significantly attenuated in olprinone -treated mice in comparison to SCI animals. The assay was carried out by using Optilab Graftek software on a Macintosh personal computer (CPU G3-266).

To test whether olprinone may modulate the inflammatory process, we analyzed the spinal cord tissue levels of pro-inflammatory cytokines. A substantial increase in TNF-α and IL-1β production was found in spinal cord tissues samples collected from SCI mice 24 hours after SCI (Figure 4A and B, respectively). Spinal cord levels of TNF-α and IL-1β were significantly attenuated by the intraperitoneal injection of olprinone (Figure 4A and B, respectively). In addition, spinal cord sections were also taken at 24 h after SCI to determine the expression of TNF-α and IL-1-β by immunohistological staining. There was no staining for TNF-α and IL-1-β in spinal cord obtained from the sham mice (Figure 4 C and F, respectively). A substantial increase in TNF-α (Figure 4D) and IL-1-β (Figure 4G) expression was found in inflammatory cells as well as in nuclei of Schwann cells in the white and gray matter of the spinal cord tissues collected from SCI mice 24 hours after SCI. Spinal cord expression of TNF-α (Figure 4E) and IL-1-β (Figure 4H) were significantly attenuated in olprinone treated-SCI mice in comparison to SCI animals.

### Effects of olprinone on nitrotyrosine formation, and PAR formation after SCI

**Figure 5:**
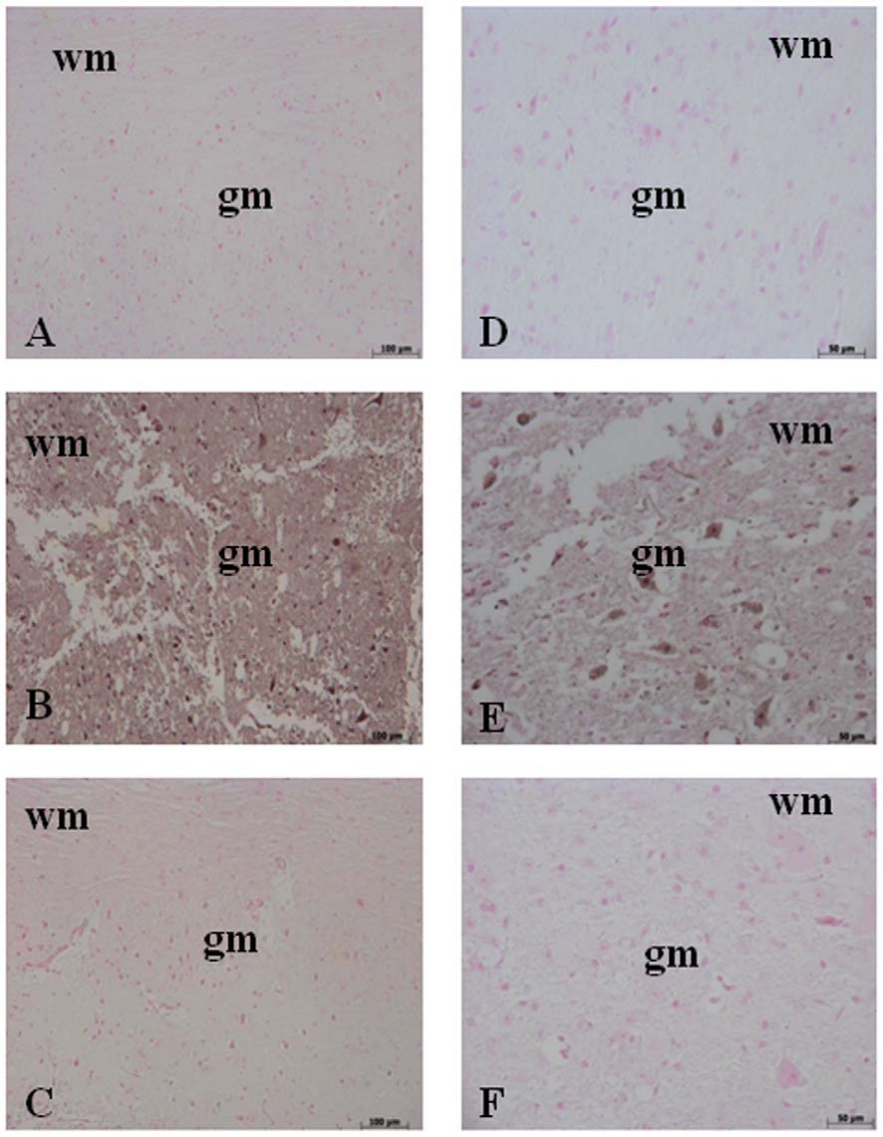
Effects of olprinone on nitrotyrosine formation and PAR. Sections obtained from SCI animals demonstrate positive staining for nitrotyrosine (**B**) mainly localized in inflammatory, in Schwann cells in the white and gray matter. Olprinone treatment (0,2 mg/kg, 1 and 6 h after SCI induction) reduced the degree of positive staining for nitrotyrosine (**C**) in the spinal cord. PAR expression, as an indicator of *in vivo* PARP activation, revealed the occurrence of positive staining localized in nuclei of Schwann cells in the white and gray matter of the spinal cord tissues from mice subjected to SCI (E). Olprinone treatment reduced the degree of positive staining for PAR (F) in the spinal cord. Figures are representative of at least 3 experiments performed on different experimental days.

Twenty-four hours after SCI, nitrotyrosine, a specific marker of nitrosative stress, was measured by immunohistochemical analysis in the spinal cord sections to determine the localization of *“peroxynitrite formation”* and/or other nitrogen derivatives produced during SCI. Spinal cord sections from sham-operated mice did not stain for nitrotyrosine (Figure 5A), whereas spinal cord sections obtained from SCI mice exhibited positive staining for nitrotyrosine (Figure 5B; 6.80±0.13, p<0.01 vs. Sham, n = 5). The positive staining was mainly localized in inflammatory cells as well as in nuclei of Schwann cells in the white and gray matter of the spinal cord tissues. Olprinone treatment reduced the degree of positive staining for nitrotyrosine (Figure 5C, 0.10±0.07 vs. 6.80±0.13, p<0.01 vs. SCI, n = 5) in the spinal cord.

In our study, immunohistochemistry for PAR, as an indicator of *in vivo* PARP activation, revealed the occurrence of positive staining for PAR localized in nuclei of Schwann cells in the white and gray matter of the spinal cord tissues from mice subjected to SCI (Figure 5E, 6.50±0.14, p< vs. Sham, n = ). Olprinone treatment reduced the degree of positive staining for PAR (Figure 5F, 0.29±0.09 vs. 6.50±0.14, p<0.01 vs. SCI, n = 5) in the spinal cord.

### Effect of olprinone on IκB-α degradation, and NF-κB p65 activation

We evaluated the degradation of the inhibitor of NF-κB, IκB-α, and nuclear NF-κB p65 by Western Blot analysis to investigate the cellular mechanisms by which treatment with olprinone may attenuate the development of SCI.

**Figure 6:**
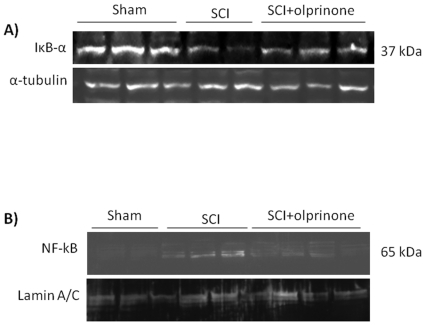
Effects of PDE III inhibition on IκB-α degradation, and nuclear NF-κB p65. By Western Blot analysis, a basal level of IκB-α was detected in the spinal cord from sham-operated animals, whereas IκB-α levels were substantially reduced in SCI mice. Olprinone treatment (0,2 mg/kg, 1 and 6 h after SCI induction) prevented the SCI-induced IκB-α degradation (**A**). In addition, SCI caused a significant increase in nuclear NF-κB p65 compared to the sham-operated mice (**B**). Olprinone treatment significantly reduced NF-κB p65 expression as shown in **B.** α-tubulin and lamin A/C were used as internal control for cytosolic and nuclear extract, respectively. A representative blot of lysates obtained from each group is shown. The relative expression of the protein bands from three separated experiments was standardized for densitometric analysis to housekeeping genes.

A basal level of IκB-α was detected in the spinal cord from sham-operated animals, whereas IκB-α levels were substantially reduced in SCI mice (Figure 6A; 5.77±0.8 vs. 11.80±1.01, p<0.01 vs. Sham; n = 5). Olprinone administration prevented the SCI-induced IκB-α degradation (Figure 6A; 9.79±0.5 vs. 5.8±0.9, p<0.05 vs. SCI; n = 5). Moreover, NF-κB p65 levels in the nuclear fractions from spinal cord tissue were also significantly increased at 24 h after SCI compared to the sham-operated mice (Figure 6B; 3.66±0.2 vs. 0.10±0.04, p<0.001 vs. Sham; n = 5). Olprinone treatment significantly reduced the levels of NF-κB p65 as shown in Figure 6B (1.49±0.41 vs. 3.67±0.2, p<0.01 vs. SCI; n = 5).

### Olprinone modulates the induction of phospho-ERK and phospho-P38 after SCI

**Figure 7:**
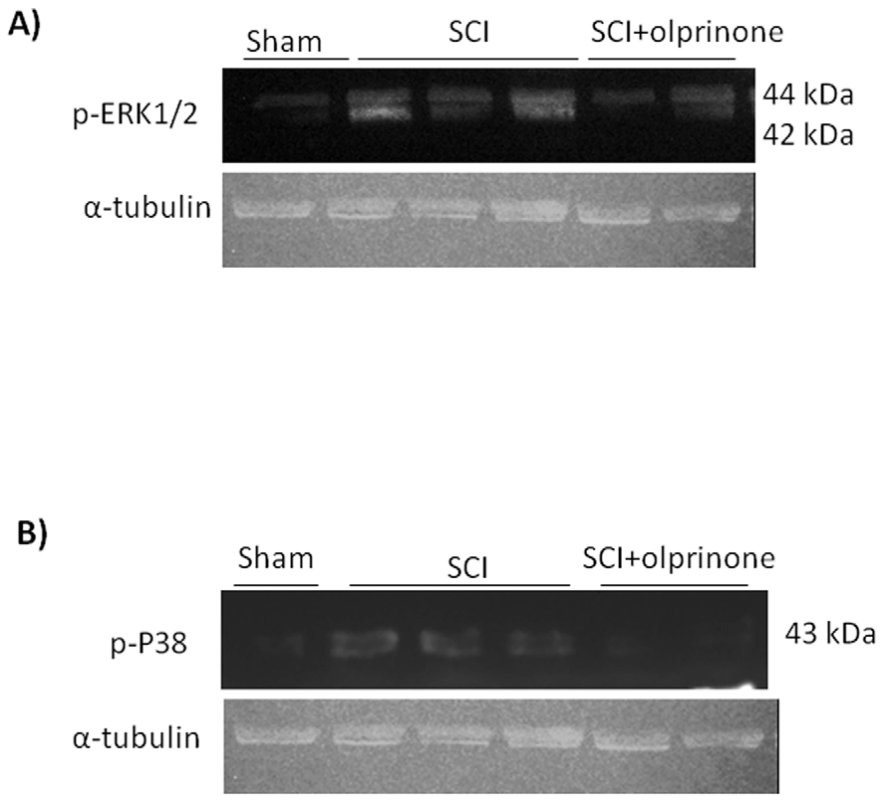
Effect of olprinone on activated kinases. The spinal cord extract were immunoblotted for the dual-phosphorylated form of ERK and for phosphor-P38. pERK1/2 is upregulated in injured mice as compared to sham-operated mice (**A**). Spinal cord levels of pERK1/2 were significantly attenuated in olprinone (0,2 mg/kg)-SCI treated mice in comparison to SCI animals (**A**). Similarly, the treatment with olprinone significantly reduced the SCI-induced increase of the expression of phospho-P38 (**B**).

We also evaluated the level of phosphorylated ERK1/2 which results in expression of pro-inflammatory genes mediating the inflammatory responses characteristic of SCI. The activation of MAPK pathways in particular the phosphorylation of ERK1/2 expression was investigated by Western blot in spinal cord homogenates at 24 h after SCI. A significant increase in pERK1/2 levels were observed in SCI mice (Figure 7A; 6.78±0.7 vs. 1.33±0.2, p<0.01 vs. Sham; n = 5). The treatment of mice with olprinone significantly reduced the level of pERK1/2 (Figure 7A; 3.21±3.2 vs. 6.78±0.7, p<0.01 vs. SCI; n = 5). In addition, at 24 h after SCI, the activation of phospho-P38 in spinal cord homogenates was investigated by Western blot. A significant increase in phospho-P38 (Figure 7B; 5.97±0.6 vs. 0.36±0.1, p<0.01 vs. Sham; n = 5) levels were observed in the spinal cord from mice subjected to SCI. On the contrary, olprinone treatment prevented the SCI-induced (Figure 7B; 2.40±0.3 vs 5.97±0.6, p<0.01 vs. SCI; n = 5) expression of these kinases.

### Effects of olprinone on FasL expression and on S100β Immunoreactive profiles in spinal cord after injury

**Figure 8:**
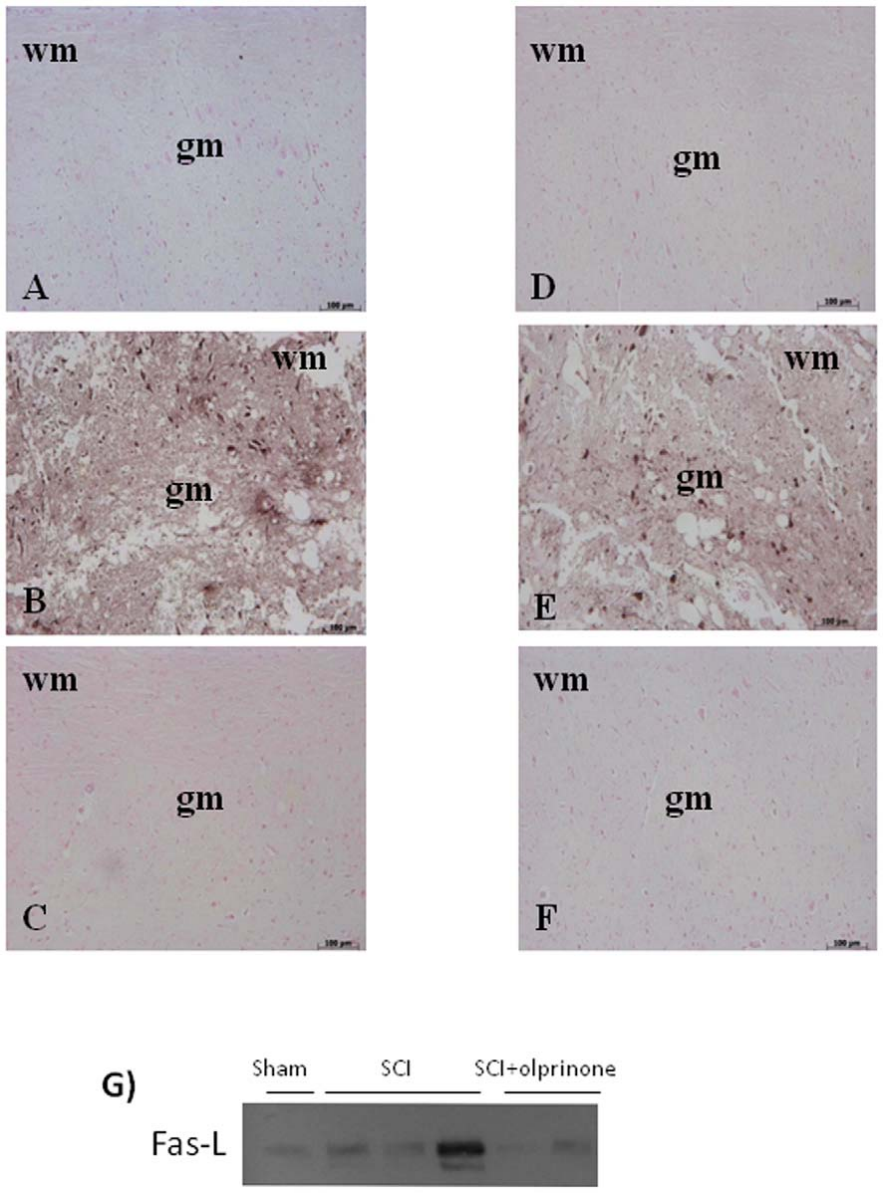
Effect of olprinone on immunohistochemical localization and expression of Fas-ligand. At 24 hr after SCI a substantial increase in Fas-ligand expression (**B**) was found in inflammatory cells, in nuclei of Schwann cells in wm and gm of the spinal cord tissues. Spinal cord Fas-ligand was significantly attenuated in olprinone-SCI treated mice in comparison to SCI animals (**C**). Moreover, SCI increased the number of S100β immunoreactive astrocytes in the white and grey matters of the spinal cord at caudal levels adjacent to the injury (**E**). This number of S100β immunoreactive astrocytes is significantly reduced in the spinal cord obtained from oprinone-treated SCI mice (**F**). Densitometry analysis of immunocytochemistry photographs (n = 5 photos from each sample collected from all mice in each experimental group) for Fas-ligand and S100β from spinal cord tissues was assessed. Similarly, at 24 h after SCI, the expression of Fas-ligand in the spinal cord homogenates was investigated by Western blot. A significant increased Fas-ligand (**H**) expression was observed in the spinal cord from mice subjected to SCI. On the contrary, olprinone treatment prevented the SCI-induced Fas-ligand (**H**) expression. This figure is representative of at least 3 experiments performed on different experimental days. wm: White matter; gm: gray matter.

To investigate the induction of apoptotic pathways triggered by Fas-FasL binding, immunohistological staining for FasL in the spinal cord was also determined 24 h after injury. Spinal cord sections from sham-operated mice did not stain for FasL (Figure 8A), whereas spinal cord sections obtained from SCI mice exhibited positive staining for FasL (Figure 8B, 5.00±0.1, p<0.01 vs. Sham, n = 5) mainly localized in inflammatory cells as well as in nuclei of Schwann cells. Olprinone treatment reduced the degree of positive staining for FasL in the spinal cord (Figure 8C, 0.20±0.07 vs 5.00±0.1, p<0.01 vs. SCI, n = 5). Similarly, a significant increase in FasL (Figure 8G; 6.28±0.9 vs. 0.28±0.1, p<0.01 vs. Sham n = 5) levels were observed in the spinal cord from mice subjected to SCI by Western blot. On the contrary, olprinone treatment prevented the SCI-induced FasL (Figure 8G; 2.58±0.7 vs. 6.28±0.9, p<0.01 vs. SCI n = 5) expression.

To characterize the changes in S100β in paracrine trophic mechanisms in the lesioned spinal cord, S100β immunoreactive quiescent astrocytes with a small size and thin processes were seen distributed throughout the white and gray matters of the sham-operated mice (Figure 8D). SCI in mice increased the number of S100β immunoreactive astrocytes with a large cytoplasm and thick processes, in the white and grey matters of the spinal cord at caudal levels adjacent to the injury (Figure 8E, 4.8±0.2). This number of S100β immunoreactive astrocytes is significantly reduced in the spinal cord obtained from olprinone-treated SCI mice (Figure 8F, 0.18±0.06 vs. 4.8±0.2, p<0.01 vs. SCI n = 5).

### Effects of olprinone on apoptosis in spinal cord after injury

**Figure 9:**
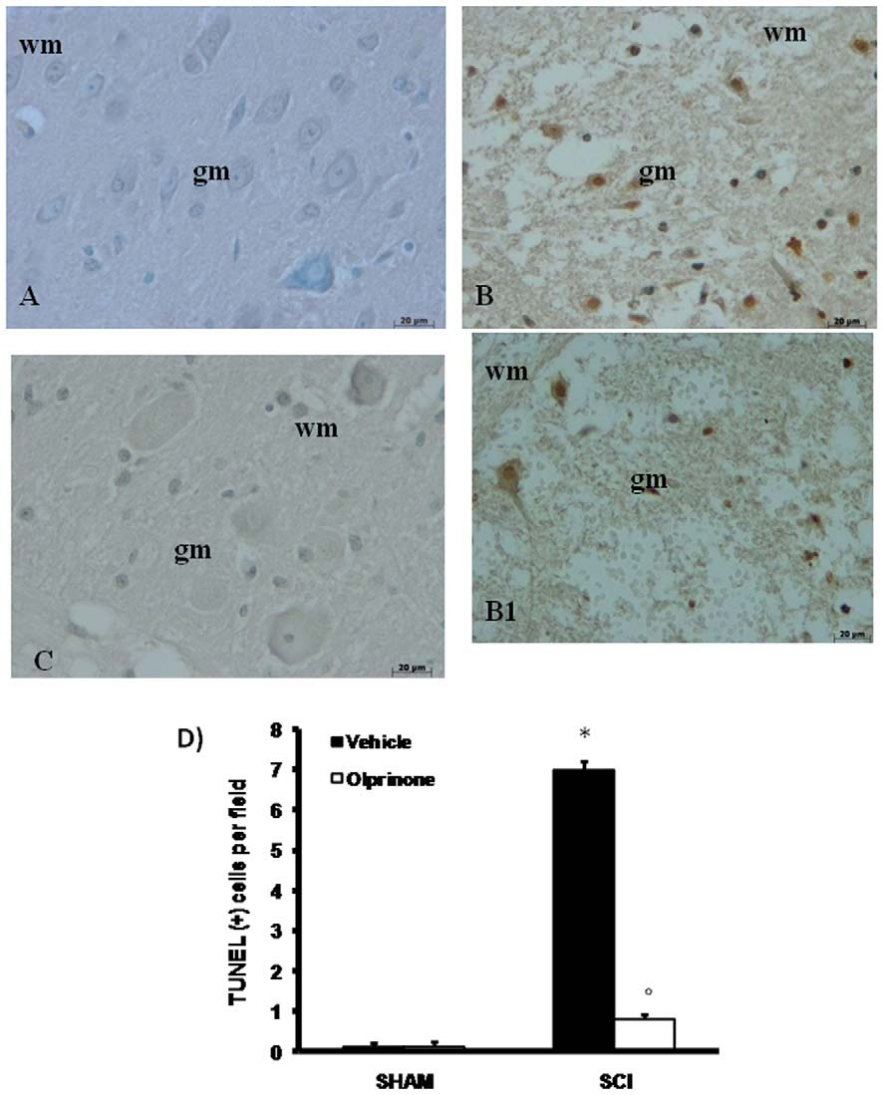
Effects of olprinone on TUNEL-like staining in the perilesional spinal cord tissue and S100β expression. At 24 h after the trauma, SCI mice demonstrated a marked appearance of dark brown apoptotic cells and intercellular apoptotic fragments (**B and B1**). In contrast, tissues obtained from mice treated with olprinone (0,2 mg/kg) demonstrated no apoptotic cells or fragments (**C**). The number of TUNEL positive cells/high-power field was counted in 5 to 10 fields for each coded slide (**D**). Figure is representative of at least 3 experiments performed on different experimental days.

To test whether spinal cord damage was associated to cell death by apoptosis, we measured TUNEL-like staining in the perilesional spinal cord tissue. Almost no apoptotic cells were detected in the spinal cord from sham-operated mice (Figure 9A). At 24 h after the trauma, tissues from SCI mice demonstrated a marked appearance of dark brown apoptotic cells and intercellular apoptotic fragments (Figure 9B B1, see positive cell count D). In contrast, tissues obtained from mice treated with olprinone demonstrated no apoptotic cells or fragments (Figure 9C, see positive cell count D).

### Western blot analysis and immunohistochemistry for Bax and Bcl-2

**Figure 10:**
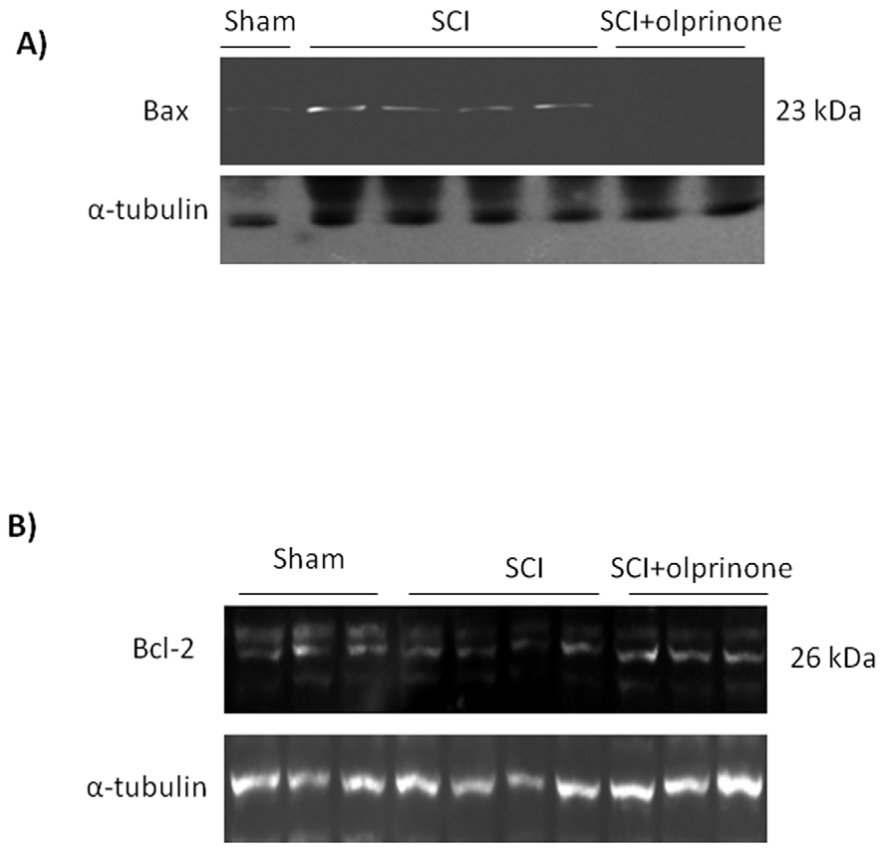
Effects of olprinone on intrinsic apoptotic pathway. SCI caused, at 24 h, an increase in Bax expression as evidenced by Western blot analysis (**A**). Olprinone treatment (0,2 mg/kg) reduced the degree of positive staining for Bax in the spinal cord (**A**). Moreover, a basal level of Bcl-2 expression was detected in spinal cord from sham-operated mice (**B**). Twenty-four hours after SCI, Bcl-2 expression was significantly reduced in spinal cord from SCI mice. Olprinone treatments (0,2 mg/kg, 1 h and 6 h after SCI induction) significantly reduced the SCI-induced inhibition of Bcl-2 expression (**B**).

At 24 h after SCI, the appearance of proteic effectors of canonical mitochondrial apoptosis, such as pro-apoptotic (Bax) and anti-apoptotic (Bcl-2) proteins, was investigated by Western blot. The balance of Bax levels were appreciably increased in the spinal cord from mice subjected to SCI (Figure 10A; 3.36±0.8 vs. 0.14±0.04, p<0.01 vs. Sham, n = 6). On the contrary, olprinone treatment prevented the SCI-induced Bax expression (Figure 10A; 0.30±0.2 vs. 3.36±0.8, p<0.01 vs. SCI n = 6).

By Western blot analysis Bcl-2 expression was also analyzed in homogenates from spinal cord of each mouse. A basal level of Bcl-2expression was detected in spinal cord from sham-operated mice (Figure 10B). Twenty-four hours after SCI, the Bcl-2 expression was significantly reduced in spinal cord from SCI mice (Figure 10B; 6.57±1.4 vs. 14.62±1.4, p<0.01 vs. Sham n = 6). Treatment of mice with olprinone significantly blunted the SCI-induced inhibition of anti-apoptotic protein expression (Figure 10B; 15.41±1.6 vs. 6.57±1.4 p<0.01 vs. SCI n = 6).

**Figure 11:**
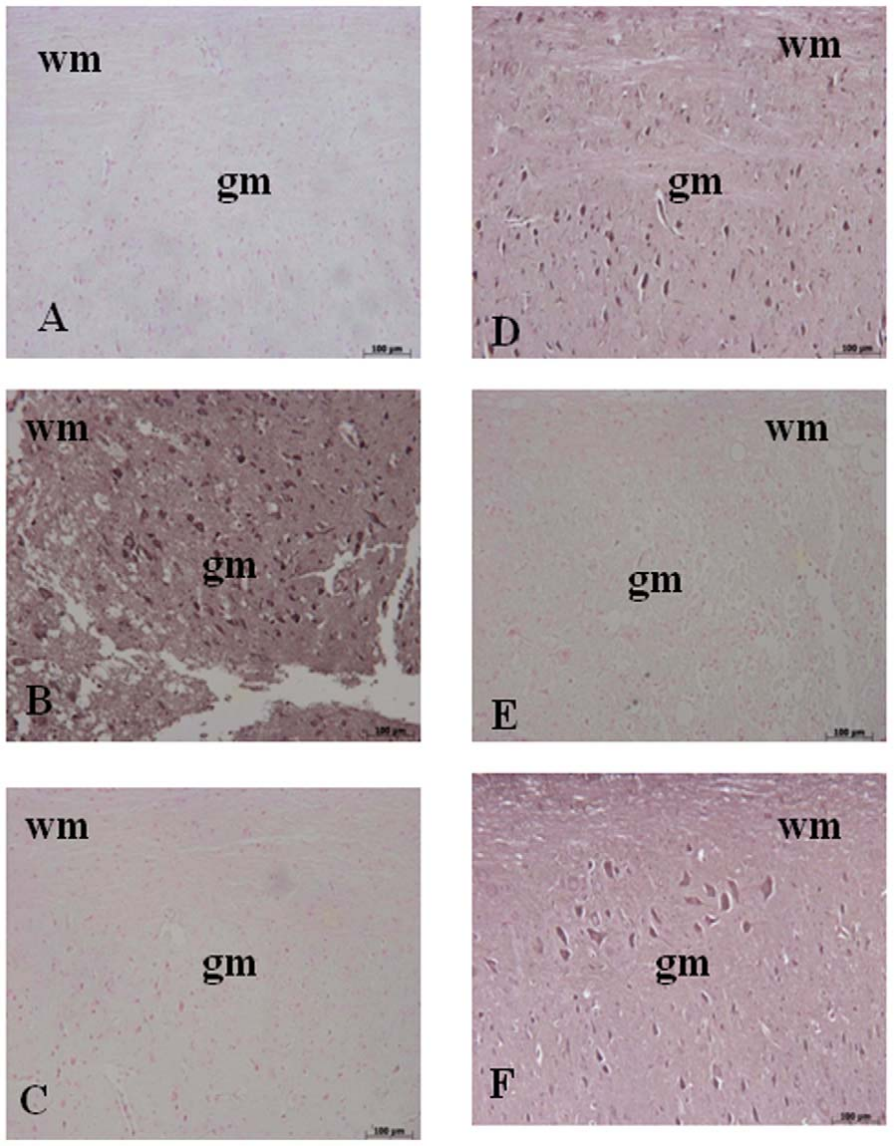
Effects of olprinone on apoptotic proteins. Staining for Bax expression was absent in Sham group (**A**). Twenty-four hours after SCI, spinal cord tissue from injured-animals showed a positive staining for Bax (**B**). Olprinone treatments (0,2 mg/kg) significantly reduced the SCI-induced Bax (**C**). On the contrary, positive staining for Bcl-2 was observed in the spinal cord tissues from sham-operated mice (**D**) while the staining was significantly reduced in SCI mice (**E**). Olprinone treatment attenuated the loss of positive staining for Bcl-2 in the spinal cord from SCI- subjected mice (**F**). Data are expressed as% of total tissue area.

Moreover, samples of spinal cord tissue taken at 24 h after SCI were also processed for immunohistological staining for Bax and Bcl-2. Spinal cord sections from sham-operated mice did not stain for Bax (Figure 11A) whereas spinal cord sections obtained from SCI mice exhibited a positive staining for Bax (Figure 11B, 4.95±0.1, p<0.01 vs. Sham, n = 5). Olprinone treatment reduced the degree of positive staining for Bax in the spinal cord of mice subjected to SCI (Figure 11C, 0.14±0.07 vs. 4.95±0.1, p<0.01 vs. SCI, n = 5). In addition, spinal cord sections from sham-operated mice demonstrated Bcl-2 positive staining (Figure 11D) while in SCI mice the staining significantly reduced (Figure 11E, 0.20±0.08 vs. 6.40±0.06, p<0.01 vs. Sham, n = 5). Olprinone treatment attenuated the loss of positive staining for Bcl-2 in the spinal cord from SCI- subjected mice (Figure 11F, 5.50±0.1 vs. 0.20±0.08, p<0.01 vs. SCI, n = 5).

## Discussion

Olprinone, a specific PDE III inhibitor, has been found to have inotropic and peripheral vasodilatory effects [4], [30]. It is well known that the substrate specificity of PDE III is cAMP > cGMP; therefore, PDE III inhibitors will increase the intracellular cAMP concentration [9]–[11]. Downstream effector proteins of cAMP and cGMP include PKA, PKG, cyclic nucleotide-gated ion channels, and cAMP-regulated guanine nucleotide exchanger factors [31].

We report here that the pharmacological inhibition of PDE III with olprinone in mice exerts a protective effect against the pathological changes caused by SCI. Thus, we propose that PDE III contributes to the pathophisiology of SCI. The mechanism of action of the PDE inhibitors has been partially elucidated and is thought to involve the enhancement of anti-inflammatory cytokines and the suppression of pro-inflammatory cytokines. cAMP-elevating agents are known to modulate cytokine production. Eigler et al. reported that cAMP elevating agents modulate immune cells by decreasing TNF-α and increasing IL-10 through PKA [32]. IL-10 has profound autoregulatory and primarily negative effects on macrophage activation [33] and macrophages are believed to play a key role during the progression of inflammation. Macrophages, as target cells, have the highest level of PDE III compared to several cell types, including lymphocytes, monocytes, and endothelial cells. Macrophages perform important functions in the removal of myelin debris and the products of neuronal degeneration following trauma, infection, or autoimmune reactions within the neuronal systems. However, the true mechanism of PDE inhibitors has not yet been fully elucidated.

Much of the damage that occurs in the spinal cord following traumatic injury is due to the secondary effects of glutamate excitotoxicity, Ca^2+^ overload, and oxidative stress, three mechanisms that take part in a spiraling interactive cascade ending in neuronal dysfunction and death [34], [35], [36], [37], [38], [39], [40].

SCI incidence and prevalence are not precisely known depending on the country. It is difficult to act on primary lesions, thus there is a critical need to develop new pharmacologic approaches for treatment of SCI.

Several lines of evidence support the potential neuroprotective effects of olprinone [41], [42].

PKA activation, caused by cAMP, is reported to lead to augmentation of p38 MAPK activity through protein tyrosine phosphatase [5]. Moreover, Xu and colleagues have clearly demonstrated in vivo an enhanced activation of ERK1/2 and p38 MAPK in the injured spinal cord after traumatic SCI persists at least for 24 h after injury [43]. Moreover, ERK1/2 and p38 MAPK signaling pathways have been found to be involved in microglia/macrophage activation [44], [45], [46]. Recently, it has been demonstrated that the elevation of cell cAMP levels inhibits NF-κB activation by targeting p38 MAPK [47]. Thus, the activity of olprinone on the cAMP levels might account for its effect on NF-κB activation, since have been showed that cAMP also activates PKA, which inhibits NF-κB [48].

NF-κB plays a central role in the regulation of many genes responsible for the generation of mediators or proteins in inflammation. These include the genes for TNF-α, IL-1β, iNOS and COX-2 [49]. In this regard, it has been well demonstrated that in SCI the expression of proinflammatory cytokines (TNF-α and IL-1β) at the site of injury regulates the precise cellular events after SCI [50], [51]. We have clearly confirmed a significant increase in TNF-α and IL-1β in SCI. On the contrary, no significant expression of TNF-α and IL-1β was observed in the spinal cord sections obtained from SCI-operated mice which received olprinone confirming that PDE III pathway play an important role in the regulation of proinflammatory cytokines. This observation is in agreement with previous studies in which have been demonstrated that olprinone treatment reduced the generation and release of proinfiammatory cytokines [52], [53], [54]. Olprinone may thus have a unique therapeutic profile, capably suppressing the earliest steps in posttraumatic inflammatory cascades.

Furthermore, we observed that treatment with olprinone abolished the expression of P-selectin and ICAM-1. These results demonstrate that inhibition of the PDEIII pathway may interrupt the interaction neutrophils and endothelial cells both at the early rolling phase mediated by P-selectin and at the late firm adhesion phase mediated by ICAM. The absence of an increased expression of the adhesion molecule in spinal cord tissue from injured mice treated with olprinone correlated with the reduction of leukocyte infiltration and with the attenuation of the spinal cord tissue damage, suggesting that it not only reduced the local inflammatory response, but also reduced leukocyte infiltration from the periphery. As neutrophils release ROS, chemokines and other key determinants of secondary tissue damage [55], the invasion of neutrophils after SCI is closely linked to the severity of secondary spinal cord injury.

Therefore, various studies have demonstrated that PARP activation after single DNA strand breakage induced by ROS plays an important role in the process of SCI [56], [57]. In this study we confirm the increase in PAR formation in the spinal cord from SCI mice and show that olprinone treatment attenuates PARP activation.

Secondary degeneration at the site of SCI appear to be due in part to apoptosis [58]. After SCI, typical post-traumatic necrosis occurred, but in addition apoptotic cells were found from 6 hours to 3 weeks after injury, especially in the spinal white matter, involving oligodendrocytes and microglia [59].

There is broad consensus in the scientific literature that SCI induces the extrinsic apoptosis pathway via Fas activation in vitro and in vivo [60], [61], [62], [63], [64]. Initiation of apoptosis provides that mitochondria and caspases engage in a self-amplifying pathway of mutual activation. Caspases might have a dual function in the apoptotic process: first, as signal-transduction molecules that act as facultative inducers of mitochondrial membrane changes, and, second, as processing enzymes that orchestrate the apoptotic phenotype [65], [66]. SCI leads to increased expression of Fas and FasL, which results in the continued activation of microglia and the inflammatory response, inducing a cascade of apoptotic cell death and extension of the initial damage [67].

Neuronal and oligodendroglial apoptosis contributes to demyelination and Wallerian degeneration, and thereby affects neuronal function and survival after SCI. In agreement with these findings, we have demonstrated a significant increase in TUNEL-positive cells in the perilesional zone following SCI. These data provide evidence that the functional neurological deficits after SCI are associated with demyelination and the loss of neurons and oligodendrocytes.

The ability of the Fas–FasL system to activate apoptotic pathways raises questions about the underlying mechanisms involved in secondary SCI. Results obtained through in vitro and in vivo experiments suggest that the Fas–FasL system also mediates the intrinsic apoptotic pathways. These findings were further supported by in vitro experiments showing that Fas was capable of transducing apoptotic signals through the mitochondrial apoptotic pathway in neuronal and glial cells after traumatic injury or exposure to the Fas-activating antibody [68]. Strategies to inhibit Fas–FasL interaction may be useful neuroprotective approaches for reducing apoptosis after SCI.

The balance of pro-apoptotic (Bax) and anti-apoptotic (Bcl-2 and Bcl-xL) proteins modulates intrinsic apoptotic cell death, thus determining the relative sensitivity of cells to apoptotic stimuli.

The anti-apoptotic proteins Bcl-2 and Bcl-xL also play an important role in controlling programmed cell death by either promoting or preventing the release of pro-apoptotic mitochondrial proteins.

Various studies have postulated that preserving Bax, a pro-apoptotic gene, plays an important role in developmental cell death [69] and in CNS injury [70]. Similarly, it has been shown that the administration of Bcl-xL fusion protein (Bcl-xL FP), (Bcl-2 is the most expressed antiapoptotic molecule in adult central nervous system) into injured spinal cords significantly increased neuronal survival, suggesting that SCI-induced changes in Bcl-xL contribute considerably to neuronal death. Based on these evidences, we have identified in SCI proapoptotic transcriptional changes, including upregulation of proapoptotic Bax and down regulation of antiapoptotic Bcl-2, by immunohystochemical staining. We report in the present study that the pharmacological inhibition of PDE III pathway by olprinone in experimental model of spinal cord trauma documents features of apoptotic cell death after SCI, suggesting that protection from apoptosis may be a prerequisite for regenerative approaches to SCI. However is not possible to exclude that anti- apoptotic effect observed after olprinone treatment it may be partially dependent on the attenuation of the inflammatory-induced damage. Further studies are needed in order to clarify these mechanisms.

S100β is an astrocytic protein generally known as a biomarker of neural cell damage. However, it has recently been suggested that inhibiting the products of S100β can be a potential therapeutic target in acute stroke patients [71]. This indicates that S100β itself might exacerbate ischemic neuronal damage, a possible mechanism of which might be propagation of neuroinflammation through astrocytic gap junction and also enhancement of NO production in the ischemic brain [72], [73]. In the present study, significantly lower levels of nitrotyrosine and S100β were detected in olprinone-treated animals. We think that the production of S100β is affected by IL-1 signals in a direct fashion and also by an unknown secondary mechanism under the complex cytokine network. The reduction of these parameters resulted in the suppression of the cytokine cycles and attenuation of the continuous oxidative stress.

Although our analysis was focused on olprinone as monotherapy for SCI, its low toxicity suggests that it may be added as adjunctive therapy to methylprednisone, minocycline, or other anti-inflammatory drugs as well. Such combination therapy may well enhance the possibility of optimal recovery of function following traumatic injury to the human spinal cord.
